# AI-enhanced assessment of psychological resilience: development and validation of a multidimensional psychological model in vocational college students

**DOI:** 10.3389/fpsyg.2026.1773434

**Published:** 2026-03-03

**Authors:** Jiajia Xu, Yuanlin Cui, Ze Zhao, Hui Yang, Yanjie Yang, Xiao Huang, Mazni Mustapha

**Affiliations:** 1 Faculty of Psychology and Social Work, Universiti Malaysia Sabah, Kota Kinabalu, Sabah, Malaysia; 2 Nanchong Vocational and Technical College, Nanchong, Sichuan, China; 3 Sichuan University of Arts and Science, Dazhou, Sichuan, China

**Keywords:** machine learning, psychological resilience, psychometric validation, risk stratification, vocational college students

## Abstract

Accurate resilience evaluation is important to help vocational college students cope with transitional stress. This study developed and validated a multidimensional resilience framework using a “dual-track” design (*N* = 1,588). Psychometric analyses (Track A) revealed a robust three-factor structure with tenacity, strength, and optimism. Measurement Invariance across genders was demonstrated. Using machine learning for predictive validation (Track B), it was found that the XGBoost model performed better (AUC = 0.883) in predicting low-resilience risk than the traditional logistic regression model. Interpretability analysis through SHAP highlighted sleep quality and perceived stress as key predictors aligning with stress–resource theory. AI enhanced this by incorporating psychometrics and algorithms to give an accurate and explainable method for early identification of those in need of support in educational settings.

## 1 Introduction

Recently, students’ mental health risks in higher education environments have received increasing attention. Research consensus indicates that academic stress, employment uncertainties, and lack of social support all contribute to heightened psychological distress. Psychological resilience is an essential protective factor that can weaken the impact of stress on emotional and functional impairments (Cai et al., 2023; Gosadi and Shnaimer, 2025). Longitudinal evidence illustrates that although academic pressure and mental health metrics change throughout the study period, the relationship between resilience levels and mental health metrics remains consistent. Therefore, the identification and prevention of low resilience have a lasting impact (Wang et al., 2023). In this context, mainstream practices mostly adopt two ways: first, enhancing students’ ability to cope with problems through psychological education, stress education, and positive psychology education; second, implementing early identification and support through standardized screening. Simultaneously, campus mental health services are exploring data-driven methods to optimize the efficiency of screening and resource allocation; however, the challenge remains to establish a stable, understandable, and context-appropriate psychological measurement system (Zhang et al., 2025).

Currently, mainstream resilience scales include the Multidimensional Scale of Perceived Social Support (MSPSS) (Zimet et al., 1988), the Emotion Regulation Questionnaire (ERQ) (Gross and John, 2003), Connor–Davidson Resilience Scale (CD-RISC) (Connor and Davidson, 2003) and the Brief Resilience Scale (BRS) (Smith et al., 2008). The MSPSS validated its three-factor structure—family, friends, and significant others—across three heterogeneous samples: undergraduate students, adolescent mothers, and adult psychiatric outpatients, demonstrating strong reliability, validity, and cross-population applicability. The ERQ was validated across three studies with samples of U. S. undergraduate students (*N* = 507) and community-dwelling adults (*N* = 140) to assess individual differences in the use of two emotion regulation strategies: cognitive reappraisal and expressive suppression. Regarding the CD-RISC, researchers tested the scale on various samples (including clinical and general populations) to assess its reliability (internal consistency, test–retest reliability) and validity (structural validity, discriminant validity, etc.). They confirmed that the CD-RISC possesses good psychometric properties, effectively distinguishing between clinical and non-clinical groups and showing expected correlations with other related scales (such as anxiety and depression scales). The BRS, using samples such as college students, community adults, and medical patients, established a brief, single-dimension scale containing only six items.

Most of the previous mainstream scales used ordinary undergraduate students, adults, or clinical groups as samples, and their core dimensions focused on “general stress coping” (such as resilience to setbacks). However, the evaluation and intervention of resilience among vocational college students present some unique problems. First, these students are in a transitional period of moving from academic training to skills training and from internship to employment (Su and He, 2023; Yang et al., 2025). Their stressors are context- and stage-specific, and resilience performances vary greatly among individuals. Second, the mechanisms of resilience involve key psychological resources. For instance, social support affects behavior through self-efficacy and resilience. Studies on vocational college samples have found that resilience plays an important mediating role between social support and procrastination. Third, the concept of resilience and the measurement scales used are not consistent. Different scales cover various dimensions and theoretical connotations, hindering cross-study comparisons and applications across groups. Recent systematic reviews have also pointed out that differences in concepts and methods within resilience scales can reduce the comparability of results (Antúnez et al., 2025; Ansari and Iqbal, 2025).

Moreover, the flaws in existing solutions highlight the need for such a study. Traditional scales mostly focus on scores, with little attention given to multidimensional structure or whether they measure attributes equally well for everyone, such as men and women, students of different years, or individuals in different jobs. Thus, a score could hold different meanings depending on who received it (Li and Zheng, 2025). Although certain studies show significant connections between resilience and negative events such as anxiety, depression, and stress—suggesting that resilience may help moderate some pathways through which physical activity and coping styles influence negative emotionality—the bulk of evidence remains correlational (Gong et al., 2023). These studies do not address the real problems of the risks and giving personal feedback on a college campus. While machine learning prediction tools for psychological distress among college students have emerged in recent years, showing potential for identifying high-risk people, models lacking psychological theoretical limitations and measurements are prone to producing black-box interpretations and context transfer issues. Thus, such models cannot yield mechanistic results that would effectively guide psychological support interventions (Cassaretto et al., 2024).

To address these problems, this study proposes a novel model that integrates psychological theory and data-driven techniques for developing and validating a multidimensional psychological resilience model and assessment tool for vocational college students. Previous empirical studies have emphasized the importance of resilience-related psychological resources and adaptive mechanisms among university students, highlighting the need for context-specific assessment frameworks in educational settings (Abdelrahman et al., 2025; Chye et al., 2024). The theoretical framework will be used to identify the key dimensions of psychological resilience and their underlying predictors, develop items and check content validity, and assess structural validity, reliability, and discriminant validity by conducting exploratory and confirmatory factor analyses. Equivalence testing across various student demographics ensures that the scale is accurate and consistent in vocational education settings. To validate ecological and criterion prediction validity, we will use a strict 70% training set and a 30% testing set split on the same data structure. We will employ logistic regression and several classical machine learning models to predict low resilience risks, comprehensively evaluate model discrimination, calibration, and clinical net benefit, and use SHapley Additive Explanations (SHAP) and other explainable methods to link important characteristics to psychological theories. This approach gives clear, useful help for groups, establishes a robust system to measure and present findings, and uses our discoveries to monitor students’ mental states and provide targeted support if needed.

## 2 Conceptual framework and hypotheses

### 2.1 Theory-driven predictor framework of psychological resilience

**Figure 1:**
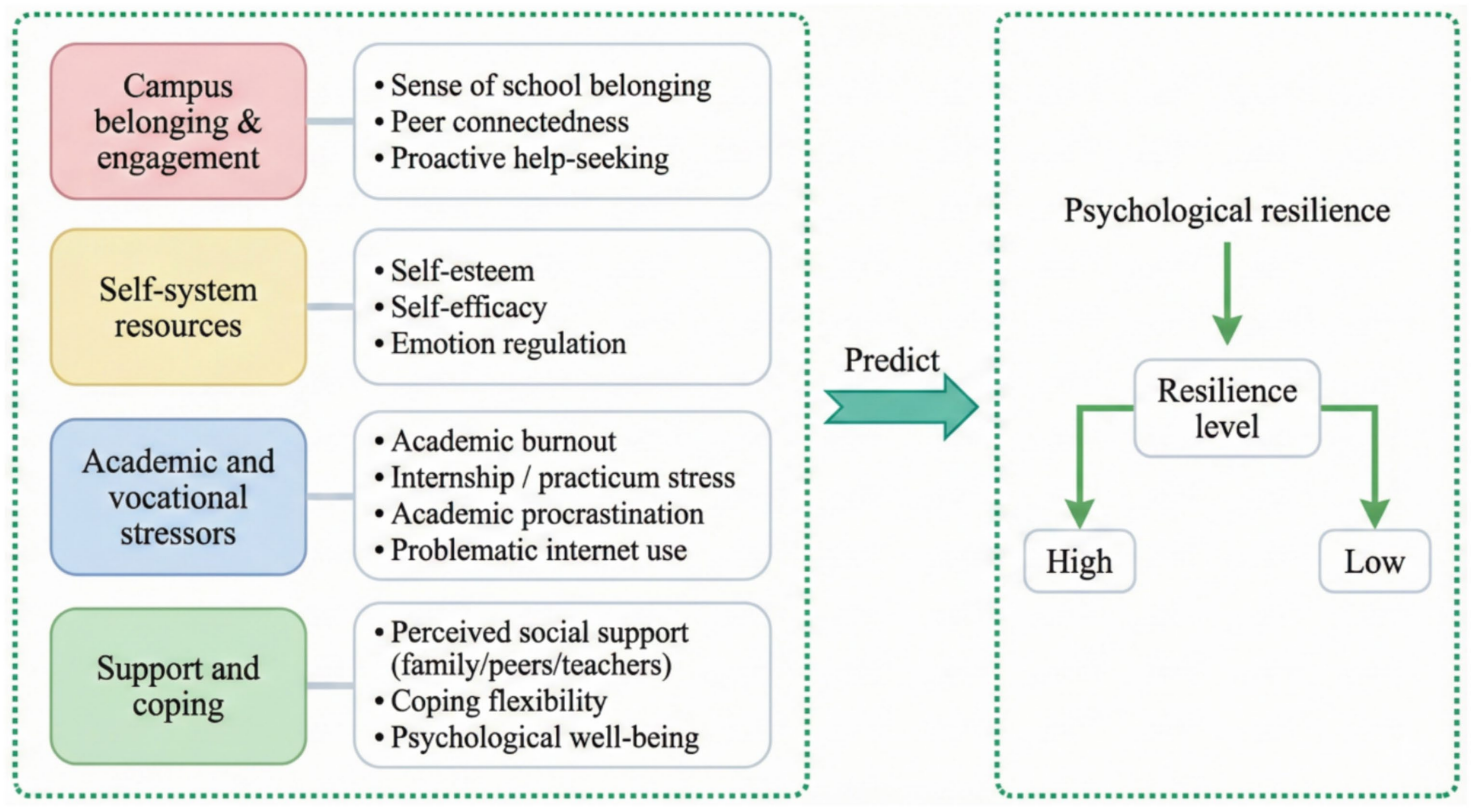
Hypothesized psychological predictors of resilience in vocational college students.

Drawing on the theoretical framework of psychological resilience, this study uses a “theory-first, variable-grounded” modeling method. It considers the mental toughness of vocational school students to be a form of adaptation that develops naturally through a developmental setting and stress. This level of resilience is not determined solely by internal personal factors but is shaped together by the school environment, academic demands, and external support systems of school. The selection predictive variables follows two principles: first, prioritizing the core psychological mechanisms that have been consistently validated in the resilience literature as closely associated with adaptive outcomes; and second, emphasizing the unique context of vocational students, including occupational learning workload, internship pressure, and employment transition stress, while also considering theoretical explanatory ability and relevance to the context. Based on this rationale, this study will combine the candidate predictors into four theoretical dimensions and form an operational hypothesis model (Figure 1) to explain and predict the level of resilience among students.

Campus belonging and participation dimensions express the quality of connections between people and their schools (Wright et al., 2022). Being part of a social group and forming peer bonds is central to socio-emotional development, providing a feeling of security and self-worth, reducing perceived threats, and thus promoting active engagement. On the contrary, a lack of belonging often leads to social withdrawal and limited resource consumption, making it easier for stress to turn into psychological exhaustion. Self-system resources focus on the internal psychological capital for resilience, such as self-esteem, self-efficacy, and emotional regulation (Xie et al., 2025). Together, these elements determine how individuals understand their skills, control their feelings, and keep trying when faced with challenges, serving as important “cushions” between tension and adaptation. Academic and occupational stressors highlight the significant risks that vocational college students encounter during their learning and professional training, including academic burnout, high-pressure internships, procrastination, and problematic internet usage (Nan, 2025). Such factors undermine individual resilience by maintaining stress and depleting self-regulation resources, increasing the risk of low resilience. Fourth, the support and coping dimension concerns protective resources such as external support systems and coping flexibility. Social support provides comfort, while coping flexibility involves the selection and adjustment of coping methods based on different types of stress (Dong et al., 2024). All these factors influence the routes taken in stress processing and how our final mental health outcomes impact our resilience.

### 2.2 The present study

This study aims to develop and validate a theory-driven psychological resilience prediction framework for vocational college students. Guided by the multidimensional psychological predictor system illustrated in Figure 1, it will systematically identify risk and protective factors associated with low psychological resilience. On this basis, multiple prediction models will be constructed and compared using a 70% training set and a 30% test set split strategy. The optimal and most interpretable model will be selected through a comprehensive evaluation of discriminative power, fit, and clinical net benefit. Furthermore, feature importance analysis will be conducted to clarify the core psychological mechanisms and directional effects, providing empirical evidence and transferable tools for the early identification, stratified management, and targeted psychological intervention of low-resilience risks among vocational college students.

#### 2.2.1 Exploratory hypotheses

Based on the “stress–resource theory” and the four-dimensional predictor framework (campus belonging and participation, self-system resources, academic and occupational stressors, support and coping), the following exploratory hypotheses are proposed:

*H1*: The three-factor structure of psychological resilience (tenacity, strength, and optimism) validated in this study will show measurement invariance not only across genders but also across different grades (freshmen, sophomores, and juniors) of vocational college students.

*H2*: Protective factors in the predictor framework (e.g., perceived social support, coping flexibility, and self-efficacy) will be negatively associated with low psychological resilience, while risk factors (e.g., academic burnout, internship stress, and problematic internet use) will be positively associated with low psychological resilience.

*H3*: Machine learning models (especially ensemble learning models like XGBoost) will outperform traditional regression models in predicting low psychological resilience, with higher AUC, accuracy, and clinical net benefit.

*H4*: Key predictors identified by SHAP analysis (e.g., sleep quality, perceived stress, social support) will align with the “stress–resource theory,” i.e., poor sleep quality and high perceived stress will increase the risk of low resilience, while strong social support will reduce this risk.

#### 2.2.2 Research questions

To further refine the study’s exploratory scope and address gaps in existing research on vocational college students’ psychological resilience, the following research questions are formulated:

*RQ1*: What is the relative importance of the four theoretical dimensions (campus belonging and participation, self-system resources, academic and occupational stressors, support and coping) in predicting vocational college students’ psychological resilience levels?

*RQ2*: Do demographic variables (e.g., major category, rural/urban origin, family income) moderate the relationship between key predictors (e.g., coping flexibility, academic pressure) and psychological resilience?

*RQ3*: To what extent can the optimal prediction model generalize to different vocational college settings (e.g., colleges focused on engineering, healthcare, or arts)?

*RQ4*: How can the model’s predictive results be translated into actionable stratified intervention strategies (e.g., targeted support for students with low sleep quality vs. high internship stress) in vocational education contexts?

## 3 Methods

### 3.1 Study design and participants

**Figure 2:**
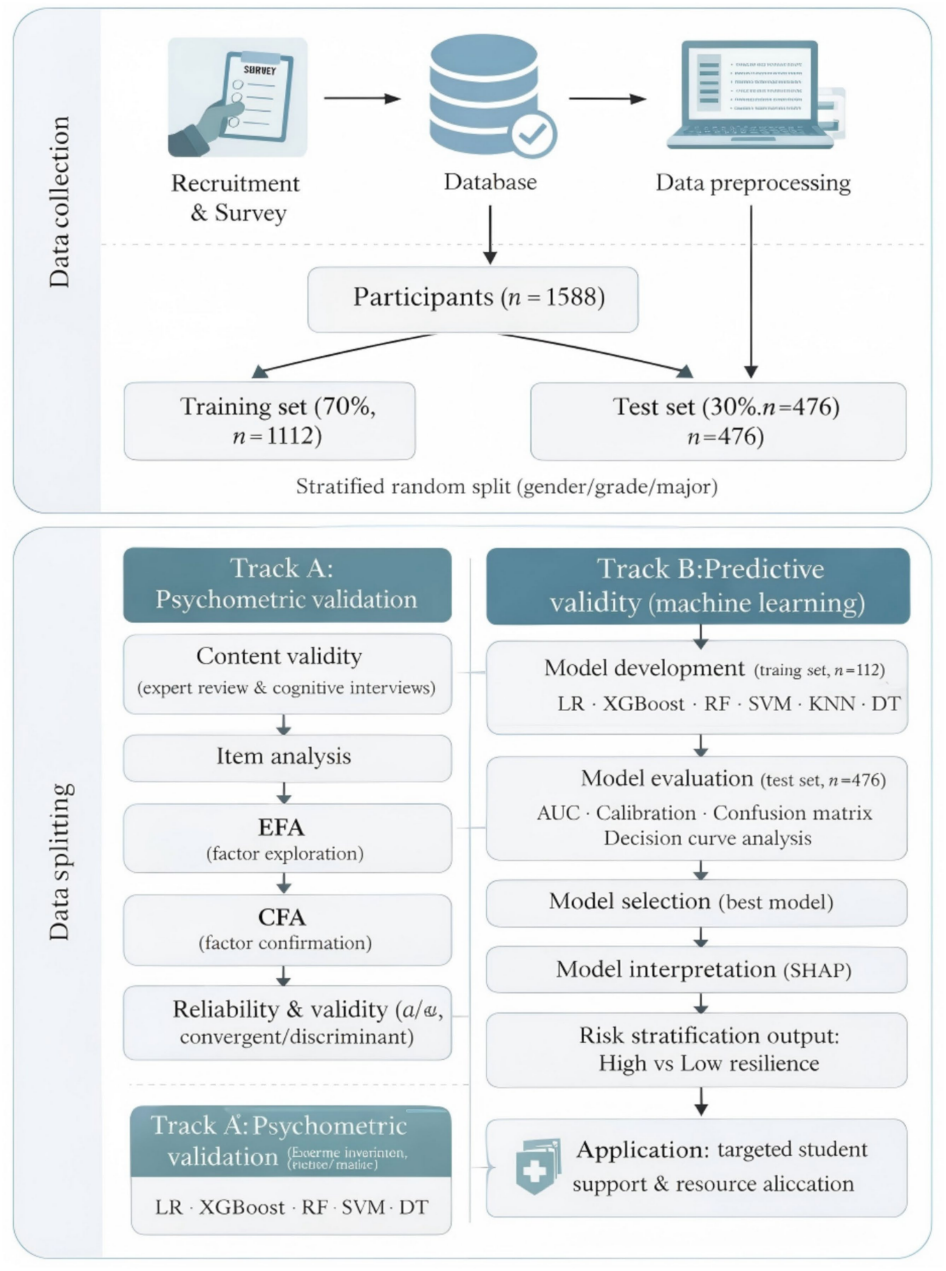
Study design and dual-track validation workflow for the multidimensional psychological resilience assessment in vocational college students (training set 70%, test set 30%).

This study conducted a cross-sectional questionnaire survey, combined with a “dual-track validation” method to establish and validate the psychological resilience evaluation model for vocational college students. The research process, as seen in Figure 2, first involved sample recruitment and scale administration, which was done in “Recruitment and Survey”. Data were entered into the database, and preprocessing was carried out (missing values and outliers were detected, variables were coded, and normalized). A total of 1,588 valid samples were included at the end. After that, the data were stratified and randomly divided (by gender, grade, and major) into a training set (70%, *n* = 1,112) and a test set (30%, *n* = 476). The training set served as the model for developing and validating it, while the testing set was used to validate the model’s ability to extend the range of application and stability.

All preprocessing steps were implemented strictly to prevent data leakage. Variables with missingness < 5% were imputed using the median (for continuous features) or mode (for categorical features) computed exclusively from the training set; variables with ≥5% missing values were excluded from analysis. For models requiring feature scaling (e.g., SVM and MLP), standardization (*z*-score normalization) was applied using the mean and standard deviation estimated only from the training set, which were then used to transform both the training and test sets. This ensured that no information from the test set influenced any aspect of preprocessing or model development.

Under “dual-track verification,” Track A is aimed at psychometric validation through a series of content validation by experts, cognitive interviews, item analysis, exploratory factor analysis (EFA), confirmatory factor analysis (CFA), and reliability/validity tests (Cronbach’s *α*, convergent validity, and discriminant validity) to ensure structural validity and measurement reliability. Track B emphasizes predictive validity by constructing and comparing multiple models (logistic regression, XGBoost, random forest, support vector machines, K-nearest neighbors, decision tree) using training data. A comprehensive evaluation is conducted on test data across discriminant power, calibration, and decision benefit metrics, with key predictors identified through SHapley Additive exPlanations (SHAP) analysis. The final output provides risk stratification results for “high/low psychological resilience” (see Figure 2).

The baseline characteristics of the samples are presented in Table 1. No significant differences were observed between the training and test sets in terms of major demographic variables (age, gender, grade, major category, place of origin, whether the child was an only child, family monthly income), academic and internship status (GPA, ranking, internship experience, part-time employment), or core psychological constructs and mental health indicators [psychological resilience, self-efficacy, perceived social support, emotion regulation, coping style, PHQ-9 depression level (Kroenke et al., 2001)] (all *p* > 0.05). This indicates that the stratified randomization achieved good intergroup comparability, providing a reliable sample foundation for subsequent model training and testing.

**Table 1:** Baseline characteristics of vocational college students in the training and test sets.

Variable	Training set (*n* = 1,112)	Test set (*n* = 476)	Total (*N* = 1,588)	*χ*^2^/*t*	*p*
Demographic characteristics					
Age (years), *M* ± SD	19.47 ± 1.23	19.51 ± 1.19	19.48 ± 1.22	0.58	0.563
Gender, *n* (%)					
Male	547 (49.2)	231 (48.5)	778 (49.0)	0.11	0.738
Female	565 (50.8)	245 (51.5)	810 (51.0)		
Academic year, *n* (%)					
First year	383 (34.4)	165 (34.7)	548 (34.5)	0.27	0.873
Second year	438 (39.4)	182 (38.2)	620 (39.0)		
Third year	291 (26.2)	129 (27.1)	420 (26.5)		
Major category, *n* (%)					
Engineering	312 (28.1)	138 (29.0)	450 (28.3)	1.84	0.606
Business and management	276 (24.8)	113 (23.7)	389 (24.5)		
Healthcare and nursing	198 (17.8)	89 (18.7)	287 (18.1)		
Arts and design	164 (14.7)	67 (14.1)	231 (14.5)		
Others	162 (14.6)	69 (14.5)	231 (14.5)		
Origin, *n* (%)					
Urban	481 (43.3)	203 (42.6)	684 (43.1)	0.09	0.769
Rural	631 (56.7)	273 (57.4)	904 (56.9)		
Only child, *n* (%)					
Yes	358 (32.2)	147 (30.9)	505 (31.8)	0.38	0.539
No	754 (67.8)	329 (69.1)	1,083 (68.2)		
Family monthly income, *n* (%)					
<3,000 RMB	243 (21.9)	107 (22.5)	350 (22.0)	2.17	0.538
3,000–6,000 RMB	478 (43.0)	197 (41.4)	675 (42.5)		
6,000–10,000 RMB	271 (24.4)	122 (25.6)	393 (24.7)		
≥10,000 RMB	120 (10.8)	50 (10.5)	170 (10.7)		
Academic and internship status					
Current GPA, *M* ± SD	3.21 ± 0.58	3.19 ± 0.61	3.20 ± 0.59	0.51	0.608
Academic ranking, *n* (%)					
Top 25%	289 (26.0)	118 (24.8)	407 (25.6)	1.52	0.677
26–50%	342 (30.8)	151 (31.7)	493 (31.0)		
51–75%	298 (26.8)	131 (27.5)	429 (27.0)		
Bottom 25%	183 (16.5)	76 (16.0)	259 (16.3)		
Internship experience, *n* (%)					
No experience	412 (37.1)	174 (36.6)	586 (36.9)	0.53	0.768
1–3 months	387 (34.8)	172 (36.1)	559 (35.2)		
>3 months	313 (28.1)	130 (27.3)	443 (27.9)		
Part-time job, *n* (%)					
Yes	478 (43.0)	198 (41.6)	676 (42.6)	0.41	0.524
No	634 (57.0)	278 (58.4)	912 (57.4)		
Psychological constructs					
Psychological resilience, *M* ± SD	67.83 ± 12.47	67.51 ± 12.89	67.74 ± 12.59	0.45	0.656
Self-efficacy, *M* ± SD	28.94 ± 5.73	28.67 ± 5.89	28.86 ± 5.77	0.83	0.405
Perceived social support, *M* ± SD	54.32 ± 11.28	53.87 ± 11.64	54.19 ± 11.38	0.71	0.479
Emotion regulation, *M* ± SD	32.58 ± 6.41	32.31 ± 6.59	32.50 ± 6.46	0.74	0.461
Positive coping style, *M* ± SD	37.16 ± 7.82	36.89 ± 7.94	37.08 ± 7.85	0.61	0.543
Negative coping style, *M* ± SD	24.73 ± 6.35	25.02 ± 6.48	24.82 ± 6.39	0.81	0.419
Mental health outcomes					
Depression (PHQ-9), *M* ± SD	8.47 ± 5.82	8.61 ± 5.94	8.51 ± 5.85	0.43	0.669
Anxiety [GAD-7 (Spitzer et al., 2006)], *M* ± SD	7.23 ± 4.96	7.38 ± 5.07	7.27 ± 4.99	0.53	0.594
Stress (PSS-10), *M* ± SD	19.82 ± 5.47	19.94 ± 5.63	19.85 ± 5.51	0.39	0.698
Academic burnout, *M* ± SD	42.36 ± 9.87	42.68 ± 10.13	42.46 ± 9.94	0.58	0.565
Life satisfaction, *M* ± SD	22.47 ± 5.38	22.29 ± 5.51	22.42 ± 5.42	0.6	0.551
Sleep quality [PSQI (Buysse et al., 1989)], *M* ± SD	7.38 ± 3.24	7.51 ± 3.31	7.42 ± 3.26	0.71	0.476
Behavioral indicators					
Physical activity (days/week), *M* ± SD	2.83 ± 1.67	2.76 ± 1.71	2.81 ± 1.68	0.74	0.457
Screen time (hours/day), *M* ± SD	5.47 ± 2.38	5.52 ± 2.43	5.48 ± 2.39	0.37	0.709
Social activities (times/month), *M* ± SD	6.82 ± 3.47	6.69 ± 3.52	6.78 ± 3.48	0.66	0.508

### 3.2 Measures and variable construction

This study constructs a candidate predictor variable system according to the “theory-driven psychological resilience prediction framework” in the following aspects: demographics, academic performance, career and internship, psychological resources, mental health indicators, and health and social behavior, to describe the psychological resilience status of vocational college students from different dimensions. The operational definitions, measurement tools, coding methods, and descriptive statistics for each variable are summarized in Table 2 and serve as the unified input specification for subsequent modeling.

**Table 2:** Operational definitions, coding schemes, and descriptive statistics of candidate predictors.

Domain	Variable	Operational definition	Measurement tool	Coding scheme	*M* ± SD/*n* (%)	Range	Skewness	Kurtosis
Demographic factors	Age	Chronological age in years	Self-report	Continuous (years)	19.48 ± 1.22	17–24	0.38	−0.42
	Gender	Biological sex	Self-report	0 = male, 1 = female	Female: 810 (51.0%)	0–1	0.04	−2.01
	Academic year	Current year of enrollment	Self-report	1 = first, 2 = second, 3 = third	1.92 ± 0.79	1–3	0.06	−1.23
	Urban–rural origin	Place of residence before college	Self-report	0 = rural, 1 = urban	Urban: 684 (43.1%)	0–1	0.28	−1.92
	Family income level	Average monthly household income	Self-report	1 = <3 k, 2 = 3-6 k, 3 = 6-10 k, 4 = ≥10 k	2.23 ± 0.91	1–4	0.37	−0.68
Academic factors	GPA	Cumulative grade point average	Academic records	Continuous (0–4 scale)	3.20 ± 0.59	1.67–4.00	−0.31	−0.18
	Major satisfaction	Satisfaction with current major	Single item (5-point Likert)	1 = very dissatisfied to 5 = very satisfied	3.67 ± 0.94	1–5	−0.52	0.13
	Academic pressure	Perceived academic stress level	4-item scale (*α* = 0.78)	Sum score (4–20)	13.47 ± 3.28	4–20	−0.14	−0.56
	Learning motivation	Intrinsic motivation for learning	6-item scale (*α* = 0.82)	Sum score (6–30)	21.43 ± 4.76	6–30	−0.29	−0.35
Career/internship factors	Internship experience	Duration of internship participation	Self-report	0 = None, 1 = 1–3 months, 2 = > 3 months	0.91 ± 0.83	0–2	0.23	−1.32
	Employment anxiety	Worry about future employment	4-item scale (*α* = 0.81)	Sum score (4–20)	12.76 ± 3.84	4–20	−0.09	−0.62
	Career clarity	Clarity of career goals and plans	5-item scale (*α* = 0.85)	Sum score (5–25)	16.82 ± 4.39	5–25	−0.18	−0.47
Psychological resources	Self-efficacy	Belief in one’s capability to succeed	General Self-Efficacy Scale (Schwarzer and Jerusalem, 1995) (10 items, *α* = 0.88)					
				Sum score (10–40)	28.86 ± 5.77	10–40	−0.24	−0.19
	Perceived social support	Availability of social support	MSPSS (Zimet et al., 1988) (12 items, *α* = 0.91)	Sum score (12–84)	54.19 ± 11.38	12–84	−0.36	0.18
	Optimism	Positive expectations for the future	LOT-R (6 items, *α* = 0.76)	Sum score (6–30)	19.34 ± 4.62	6–30	−0.28	−0.31
	Emotion regulation	Ability to manage emotional experiences	ERQ (Gross and John, 2003) (10 items, *α* = 0.84)	Sum score (10–70)	32.50 ± 6.46	10–70	0.12	0.08
	Problem-focused coping	Active problem-solving strategies	SCSQ-Active (10 items, *α* = 0.87)	Sum score (10–50)	37.08 ± 7.85	10–50	−0.35	0.17
	Self-compassion	Kind attitude toward oneself	SCS-SF (12 items, *α* = 0.83)	Sum score (12–60)	34.58 ± 7.92	12–60	−0.09	−0.34
Mental health indicators	Depressive symptoms	Severity of depressive symptoms	PHQ-9 (*α* = 0.89)	Sum score (0–27)	8.51 ± 5.85	0–27	0.64	−0.18
	Anxiety symptoms	Severity of anxiety symptoms	GAD-7 (*α* = 0.91)	Sum score (0–21)	7.27 ± 4.99	0–21	0.58	−0.29
	Perceived stress	Overall stress level	PSS-10 (Cohen et al., 1983) (*α* = 0.86)	Sum score (0–40)	19.85 ± 5.51	0–40	0.19	−0.15
	Academic burnout	Emotional exhaustion from studying	MBI-SS (15 items, *α* = 0.88)	Sum score (15–90)	42.46 ± 9.94	15–90	0.23	0.09
	Life satisfaction	Overall satisfaction with life	SWLS (5 items, *α* = 0.87)	Sum score (5–35)	22.42 ± 5.42	5–35	−0.41	0.22
Health and social behaviors	Sleep quality	Overall quality of sleep	PSQI (*α* = 0.81)	Global score (0–21)	7.42 ± 3.26	0–21	0.47	0.16
	Physical activity	Frequency of moderate-vigorous exercise	Self-report	Days per week (0–7)	2.81 ± 1.68	0–7	0.38	−0.73
	Peer relationship quality	Quality of relationships with peers	6-item scale (*α* = 0.84)	Sum score (6–30)	21.93 ± 4.58	6–30	−0.31	−0.19
	Family cohesion	Emotional bonding among family members	FACES-II (10 items, *α* = 0.86)	Sum score (10–50)	34.67 ± 7.84	10–50	−0.28	−0.21
	Romantic relationship	Currently in a romantic relationship	Self-report	0 = no, 1 = yes	Yes: 538 (33.9%)	0–1	0.66	−1.56

Demographic and academic baseline information (e.g., age, gender, grade level, place of origin, family income, and GPA) were primarily obtained through self-report or academic records and coded in continuous, ordered, or binary forms. Variables related to academic and occupational stress (e.g., academic pressure, learning motivation, employment anxiety, and career clarity) were scored using multi-item scales, with total scores reflecting individual levels (Jayaraj et al., 2025). Psychological resource variables (e.g., self-efficacy, perceived social support, optimism, emotion regulation, problem-focused coping, and self-compassion) were measured using established scales with proven reliability and validity (Lei et al., 2025). Mental health indicators (e.g., depression, anxiety, perceived stress scale, academic burnout, and life satisfaction) were used to characterize the coexistence of negative and positive psychological states. Health and social behavior variables (e.g., sleep quality, physical activity, peer relationship quality, family cohesion, romantic relationships) were employed to supplement risk and protective factors at the lifestyle and social connection levels. Overall, Table 2 demonstrates clear value ranges and interpretable distribution characteristics (providing skewness and kurtosis information), laying a reproducible foundation for variable construction in subsequent model training, comparison, and interpretive analysis.

### 3.3 Development of the psychological resilience scale

The initial item pool for the psychological resilience scale was developed through an extensive review of existing literature and aligned with the theoretical framework described in Section 2.1. To ensure content validity, a panel of five experts—including two professors specializing in psychology (with expertise in positive psychology and psychometrics), two frontline mental health practitioners from vocational colleges, and one associate professor in vocational education—reviewed the draft containing 25 initial items. Experts independently evaluated each item’s relevance, clarity, and conciseness using a 4-point scale (1 = not relevant, 4 = highly relevant). The Scale-Level Content Validity Index (S-CVI/Ave) was calculated as 0.92, indicating strong overall content validity. Items with an Item-Level CVI (I-CVI) below the recommended threshold of 0.78 were either revised or removed based on both quantitative ratings and qualitative feedback from the expert panel.

Following expert review, cognitive interviews were conducted with a purposive sample of 12 vocational college students (6 male, 6 female; representing different academic years and majors) to examine how respondents interpreted and processed the items. Using a retrospective verbal probing approach, participants completed the draft scale and were then asked to explain their understanding of each item and the rationale for their responses. Audio-recorded interviews were transcribed and analyzed thematically (Nowlin et al., 2025). Findings revealed that three items were consistently misinterpreted or perceived as ambiguous (e.g., Item 7 was frequently confused with emotional suppression rather than adaptive coping). These items were reworded to improve clarity, reduce jargon, and better reflect the intended construct. After two rounds of refinement based on cognitive interview feedback, the final 18-item scale was completed for subsequent psychometric validation.

### 3.4 Construction of outcome variables

The primary outcome variable for the predictive modeling in Track B was psychological resilience, operationalized as a continuous total score derived from the newly developed 18-item Psychological Resilience Scale (see Section 3.3). This total score was subsequently dichotomized for classification purposes (see Section 5.1).

Furthermore, the three core dimensions of psychological resilience—Tenacity, Strength, and Optimism—were constructed as subscale scores. Each subscale score was calculated as the sum of its corresponding six items, as defined by the final factor structure presented in Table 3.

**Table 3:** Item analysis and factor loading summary of the resilience scale (EFA results).

Item	CITC	CR	*M*	SD	Factor 1 tenacity	Factor 2 strength	Factor 3 optimism	*h* ^2^
Factor 1: Tenacity								
R01. I persist in pursuing my goals despite obstacles	0.647	11.83***	3.74	0.89	0.782	0.134	0.187	0.667
R02. I can stick to difficult tasks until completion	0.681	12.47***	3.68	0.92	0.814	0.162	0.143	0.713
R03. I bounce back quickly after setbacks	0.623	11.29***	3.62	0.94	0.741	0.218	0.176	0.628
R04. I maintain focus on long-term objectives	0.658	12.08***	3.71	0.87	0.793	0.147	0.198	0.687
R05. I do not give up easily when facing challenges	0.692	12.74***	3.79	0.91	0.826	0.189	0.152	0.741
R06. I can endure pressure in pursuing important goals	0.631	11.56***	3.66	0.93	0.758	0.241	0.163	0.651
Factor 2: Strength								
R07. I can control my emotions when stressed	0.618	10.94***	3.58	0.96	0.187	0.747	0.172	0.621
R08. I remain calm under pressure	0.664	11.87***	3.52	0.98	0.163	0.809	0.148	0.703
R09. I cope well with uncertainty and changes	0.637	11.41***	3.61	0.94	0.192	0.771	0.214	0.673
R10. I can manage my anxiety effectively	0.671	12.13***	3.47	1.01	0.149	0.823	0.187	0.729
R11. I maintain emotional balance in difficult times	0.643	11.62***	3.54	0.97	0.176	0.784	0.203	0.687
R12. I recover emotionally from stressful events	0.609	10.78***	3.63	0.93	0.214	0.738	0.176	0.618
Factor 3: Optimism								
R13. I believe difficulties are temporary	0.627	11.23***	3.69	0.91	0.163	0.189	0.764	0.648
R14. I see opportunities in challenging situations	0.658	11.94***	3.73	0.88	0.197	0.154	0.802	0.702
R15. I maintain hope even when things are difficult	0.641	11.58***	3.67	0.93	0.178	0.207	0.778	0.681
R16. I expect positive outcomes from my efforts	0.617	11.06***	3.76	0.86	0.213	0.163	0.751	0.638
R17. I adapt my thinking to new circumstances	0.633	11.47***	3.71	0.89	0.186	0.218	0.769	0.667
R18. I find meaning in adversity	0.594	10.51***	3.58	0.97	0.147	0.183	0.719	0.587
Eigenvalue					6.847	2.134	1.692	
% of variance					38.04	11.86	9.4	
Cumulative %					38.04	49.9	59.3	
Factor *α* coefficient					0.893	0.887	0.879	
Factor ω coefficient					0.896	0.891	0.883	

*** *p* < 0.001.

The other psychological constructs mentioned in the analysis (i.e., self-efficacy, social support, emotion regulation, depression, anxiety, stress, and academic burnout) were not constructed by the authors but were measured directly using established, validated scales. Their total scores, as provided by the respective instruments listed in Table 2, were used as predictor variables in the machine learning models and for correlation analyses in the psychometric validation (Track A). Specifically:

Self-efficacy: Total score from the General Self-Efficacy Scale (GSES).

Social support: Total score from the Multidimensional Scale of Perceived Social Support (MSPSS).

Emotion regulation: Total score from the Emotion Regulation Questionnaire (ERQ).

Depression: Total score from the Patient Health Questionnaire-9 (PHQ-9).

Anxiety: Total score from the Generalized Anxiety Disorder-7 (GAD-7).

Stress: Total score from the Perceived Stress Scale-10 (PSS-10).

Academic burnout: Total score from the Maslach Burnout Inventory–Student Survey (MBI-SS).

In summary, the resilience-related dimensions (Tenacity, Strength, Optimism, and Total Resilience) are composite scores from our new scale, while the other listed constructs are direct total scores from pre-existing, widely used measurement tools.

## 4 Track A: psychometric validation

### 4.1 Item analysis and exploratory factor analysis

To validate the structural validity and item quality of the psychological resilience scale in vocational college students, this study first conducted item analysis, followed by exploratory factor analysis (EFA). Prior to EFA, the suitability of the data for factor analysis was examined. The Kaiser–Meyer–Olkin (KMO) measure of sampling adequacy was 0.932, indicating excellent sample adequacy (well above the recommended threshold of 0.80). Bartlett’s test of sphericity was highly significant (*χ*^2^ = 3876.42, df = 153, *p* < 0.001), confirming that the correlation matrix was appropriate for factor analysis.

An EFA was performed using principal axis factoring (PAF) as the extraction method. Given the theoretical expectation that the latent factors of psychological resilience are intercorrelated, an oblique rotation (Promax) was applied to allow for correlated factors and enhance interpretability. Initial eigenvalues and the scree plot supported a three-factor solution, which together explained 62.8% of the total variance.

The final factor structure demonstrated strong factorial clarity. Items were retained only if they met the following criteria: (1) primary factor loading ≥ 0.40, and (2) no substantial cross-loadings—defined as a secondary loading < 0.30 and a difference between the highest and second-highest loadings ≥ 0.20. All 18 items satisfied these conditions, with each loading predominantly on one of the three factors and showing negligible cross-loadings on others.

As shown in Table 3, the corrected item-total correlation (CITC) values ranged from 0.52 to 0.78, and all critical ratio (CR) values were statistically significant (*p* < 0.001). These results indicate that the items possess high discriminative power and internal consistency, effectively differentiating among students with varying levels of psychological resilience.

### 4.2 Confirmatory factor analysis and measurement invariance

To further validate the scale’s structural validity, Confirmatory Factor Analysis (CFA) was conducted using the test set derived from the machine learning analysis described in the preceding section. As shown in Table 4, the three-factor model achieved a satisfactory fit (*χ*^2^/df = 3.126, RMSEA = 0.041, SRMR = 0.033, CFI = 0.968, TLI = 0.962), demonstrating that the’ resilience–power–optimism’ framework effectively explains the psychological resilience data of vocational college students and supports the structural framework derived from the earlier exploratory factor analysis (EFA).

**Table 4:** Model fit indices and measurement invariance tests across groups (CFA and invariance results).

Model	*χ* ^2^	df	*χ*^2^/df	RMSEA [90% CI]	SRMR	CFI	TLI	Δ*χ*^2^	Δdf	*p*	ΔCFI	ΔRMSEA
Total sample (*N* = 1,588)												
Three-factor model	412.584	132.000	3.126	0.041 [0.038, 0.045]	0.033	0.968	0.962					
Measurement invariance by gender (male *n* = 778 vs. female *n* = 810)												
M1: configural invariance	598.241	264.000	2.266	0.043 [0.039, 0.047]	0.036	0.965	0.959					
M2: metric invariance	619.582	279.000	2.221	0.042 [0.038, 0.046]	0.038	0.964	0.961	21.341	15.000	0.126	−0.001	−0.001
M3: scalar invariance	645.117	294.000	2.194	0.042 [0.038, 0.045]	0.041	0.963	0.962	25.535	15.000	0.043	−0.001	0.000

Building on this foundation, the study further examined the measurement invariance of the scale across gender groups (*n* = 778 males, *n* = 810 females). The results demonstrated that the configural invariance model (M1) showed a good fit. In the measurement invariance test (M2) with equal factor loadings, the changes in ΔCFI and ΔRMSEA were minimal (see Table 4), indicating comparable measurement units and structural meanings between genders. The scalar invariance test (M3) showed large *χ*^2^ differences but small incremental changes in fit indices (ΔCFI ≈ − 0.001, ΔRMSEA ≈ 0.000; see Table 4). These findings together imply that there is high cross-group comparability of the scale, making it appropriate to conduct subsequent comparisons and predictions of students’ psychological resilience levels across different genders.

### 4.3 Reliability and construct validity evidence

This study provides evidence for the quality of the scale through two dimensions: internal consistency and construct correlation. First, the three dimensions and total scores all demonstrate good internal consistency. Cronbach’s *α* and McDonald’s *ω* for resilience, strength, optimism, and total resilience are all at a high level, as shown in Table 5, indicating that the psychological resilience scale is stable and reliable in measuring vocational college students.

**Table 5:** Descriptive statistics and correlations among resilience dimensions and related psychological constructs.

Variable	*M*	SD	α	ω	1	2	3	4	5	6	7	8	9	10	11
1. Tenacity	22.47	4.83	0.847	0.851	1										
2. Strength	23.18	4.67	0.823	0.828	0.624***	1									
3. Optimism	21.92	5.14	0.864	0.869	0.587***	0.613***	1								
4. Total resilience	67.74	12.59	0.912	0.915	0.853***	0.862***	0.847***	1							
5. Self-efficacy	28.86	5.77	0.883	0.887	0.516***	0.542***	0.558***	0.613***	1						
6. Social support	54.19	11.38	0.908	0.912	0.473***	0.498***	0.521***	0.567***	0.489***	1					
7. Emotion regulation	32.5	6.46	0.841	0.846	0.448***	0.467***	0.493***	0.524***	0.537***	0.441***	1				
8. Depression	8.51	5.85	0.893	0.897	−0.487***	−0.512***	−0.563***	−0.584***	−0.523***	−0.478***	−0.502***	1			
9. Anxiety	7.27	4.99	0.908	0.911	−0.463***	−0.487***	−0.524***	−0.551***	−0.496***	−0.443***	−0.487***	0.781***	1		
10. Stress	19.85	5.51	0.857	0.862	−0.529***	−0.547***	−0.571***	−0.607***	−0.548***	−0.512***	−0.531***	0.692***	0.713***	1	
11. Academic burnout	42.46	9.94	0.882	0.886	−0.441***	−0.468***	−0.493***	−0.521***	−0.487***	−0.423***	−0.459***	0.637***	0.628***	0.684***	1

*** *p* < 0.001.

Second, the correlation analysis results support its construct validity. As shown in Table 5, the three dimensions of psychological resilience exhibit significant positive correlations with each other and with the total score, indicating that the three-dimensional structure is both interrelated and collectively reflects overall psychological resilience. Meanwhile, total resilience and its dimensions show significant positive correlations with self-efficacy, social support, and emotional regulation, aligning with the theoretical expectation of “psychological resources promoting resilience.” Conversely, they demonstrate significant negative correlations with depression, anxiety, stress, and academic burnout, reflecting the protective role of psychological resilience against negative mental health outcomes and learning exhaustion. Overall, these systematic correlations in both direction and strength provide robust support for the scale’s convergent validity and criterion-related validity (see Table 5).

## 5 Track B: predictive validity assessment (machine learning as a tool)

### 5.1 Model development in the training set (70%)

Prior to model development, the continuous total score of psychological resilience was dichotomized to define the binary outcome of “low psychological resilience.” To identify students at the greatest risk and most in need of targeted support within our sample, we employed a percentile-based cutoff derived from the empirical distribution of resilience scores in the full cohort. Specifically, participants scoring at or below the 25th percentile (P25 = 59.0) were classified as having “low psychological resilience,” while those above this threshold were categorized as having “normal/high psychological resilience.” This approach aligns with common practice in educational and psychological screening studies that aim to flag the most vulnerable subgroup (typically the lowest quartile) for early intervention.

In the total sample (*N* = 1,588), the prevalence of the low-resilience group was 25.0% (*n* = 397). The dataset was then partitioned into training and testing sets using stratified random sampling based on gender, academic year, and major discipline to preserve the original distribution of key covariates. Consequently, the prevalence of low psychological resilience remained stable across subsets: 25.1% (*n* = 279) in the training set (*n* = 1,112) and 24.8% (*n* = 118) in the hold-out test set (*n* = 476), with no statistically significant difference between groups (*χ*^2^ = 0.04, *p* = 0.841). This balanced and stable class distribution ensures robust model training and valid performance evaluation, minimizing bias due to data splitting.

Using this well-defined binary outcome, we developed and compared multiple machine learning models—including logistic regression, random forest, support vector machine, and XGBoost—on the training set (70% of the total sample). Hyperparameter tuning was performed via five-fold cross-validation within the training set to optimize predictive performance while mitigating overfitting.

To avoid optimistic bias in performance estimation, all model hyperparameters were optimized using a nested cross-validation framework within the training set. Specifically, an outer five-fold cross-validation loop was used for unbiased performance evaluation, while an inner stratified five-fold cross-validation loop was employed for hyperparameter selection. We adopted Bayesian optimization to efficiently search over predefined hyperparameter spaces. For example, the search space for XGBoost included: n_estimators ∈ [100, 500], max_depth ∈ {3, 4,…, 10}, learning_rate ∈ [0.01, 0.3], and subsample ∈ [0.6, 1.0]. The final model was retrained on the full training set using the best hyperparameters identified by the inner loop and evaluated on the held-out test set.

### 5.2 Model performance in test set (30%)

In the independent test set (30%), all candidate models were subjected to consistent evaluation and cross-reference (Table 6). XGBoost had the best overall performance, achieving an AUC of 0.883 (95% CI: 0.854–0.908), an accuracy of 0.826, an F1-score of 0.800, and a balanced accuracy of 0.825. It also had the lowest Brier score of 0.116, demonstrating the best balance between distinguishing well and maintaining good probability calibration (Table 6). Random forest and neural network followed with AUCs of 0.871 and 0.864, respectively, while support vector machine and logistic regression performed slightly lower. Decision tree and k-nearest neighbors exhibited relatively weaker performance (Table 6).

**Table 6:** Predictive performance of machine learning models for low psychological resilience in the test set (30%).

Model	AUC	95% CI	Accuracy	Sensitivity	Specificity	PPV	NPV	F1-Score	Balanced accuracy	Brier score
Logistic regression	0.847	0.814–0.878	0.793	0.768	0.811	0.741	0.829	0.754	0.790	0.137
Random forest	0.871	0.841–0.899	0.812	0.794	0.826	0.769	0.843	0.781	0.810	0.124
XGBoost	0.883	0.854–0.908	0.826	0.812	0.837	0.789	0.856	0.800	0.825	0.116
Support vector machine	0.858	0.826–0.887	0.804	0.781	0.821	0.758	0.838	0.769	0.801	0.131
K-nearest neighbors	0.824	0.789–0.857	0.776	0.743	0.801	0.718	0.817	0.730	0.772	0.149
Decision tree	0.792	0.754–0.827	0.751	0.709	0.783	0.687	0.798	0.698	0.746	0.168
Neural network	0.864	0.833–0.892	0.809	0.788	0.824	0.764	0.841	0.776	0.806	0.127

The symbol *** indicates statistical significance at the 0.001 level.

**Figure 3:**
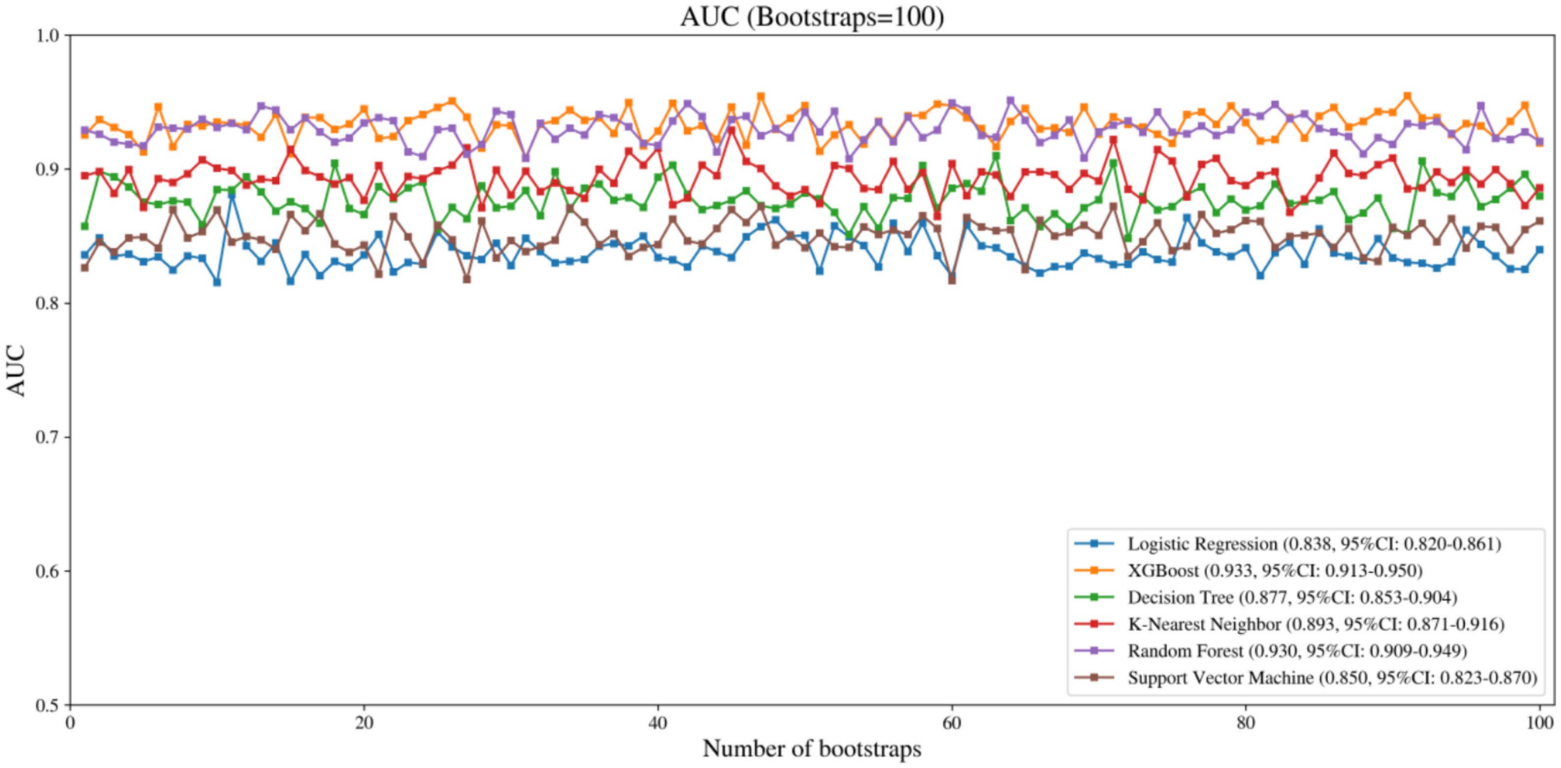
Bootstrapped AUC distributions (100 resamples) for predicting low psychological resilience across models.

In terms of robustness, AUC distributions from the 100 bootstrap resampling trials show that both XGBoost and Random Forest have consistently high and stable AUCs across multiple samplings (Figure 3), indicating they are better at generalizing.

**Figure 4:**
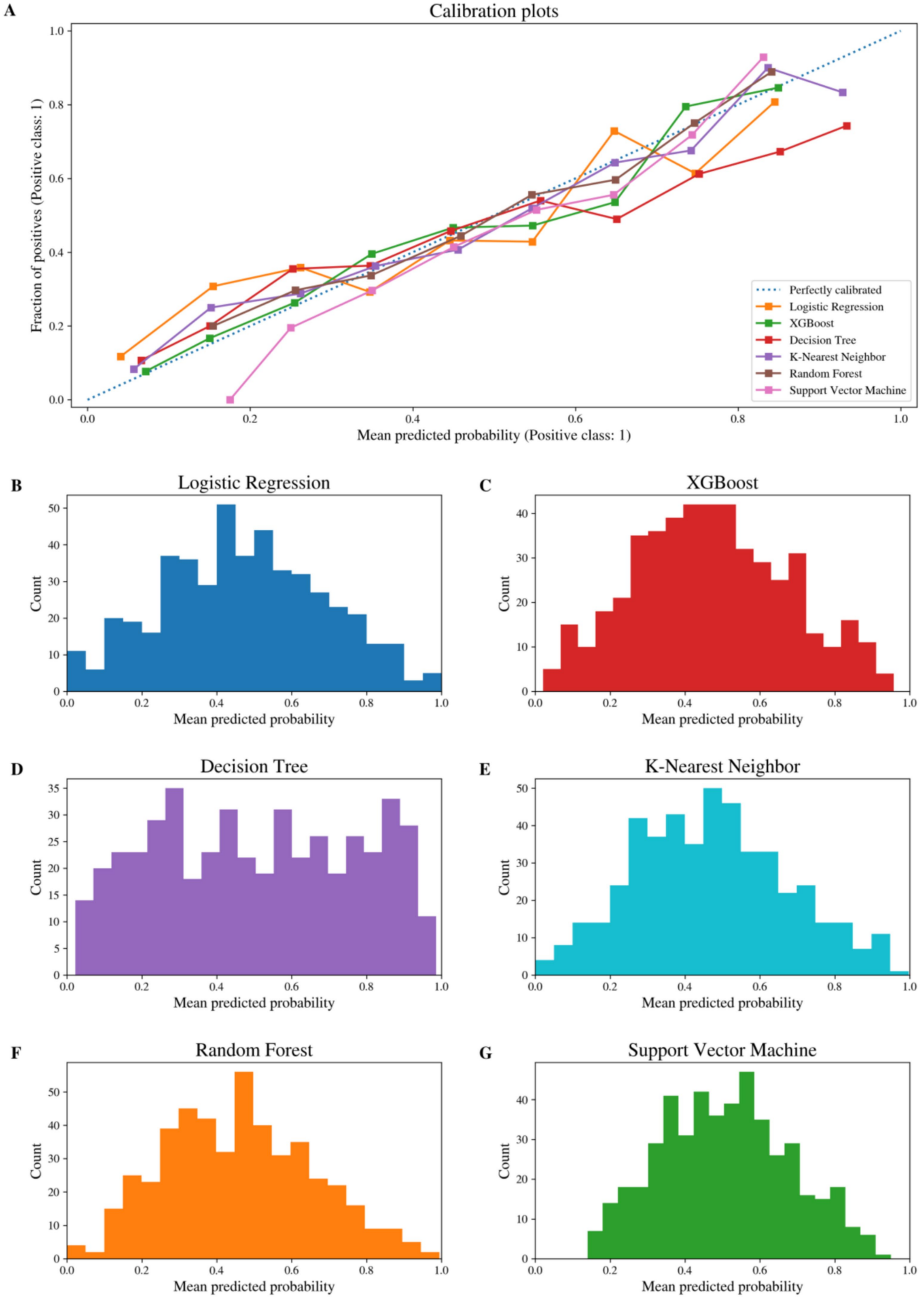
Calibration performance of prediction models for low psychological resilience. **(A)** Calibration curves comparing predicted probabilities and observed outcomes across models (logistic regression, XGBoost, decision tree, K-nearest neighbor, random forest, and support vector machine). **(B–G)** Distributions of mean predicted probabilities for each model: **(B)** Logistic Regression, **(C)** XGBoost, **(D)** Decision Tree, **(E)** K-Nearest Neighbor, **(F)** Random Forest, and **(G)** Support Vector Machine.

Calibration level refers to the discrepancies between predicted probabilities and actual incidence rates from the models based on calibration curves. Overall, the calibration of the integrated model is closer to the ideal diagonal than some models in the medium-to-high risk range (Figure 4).

**Figure 5:**
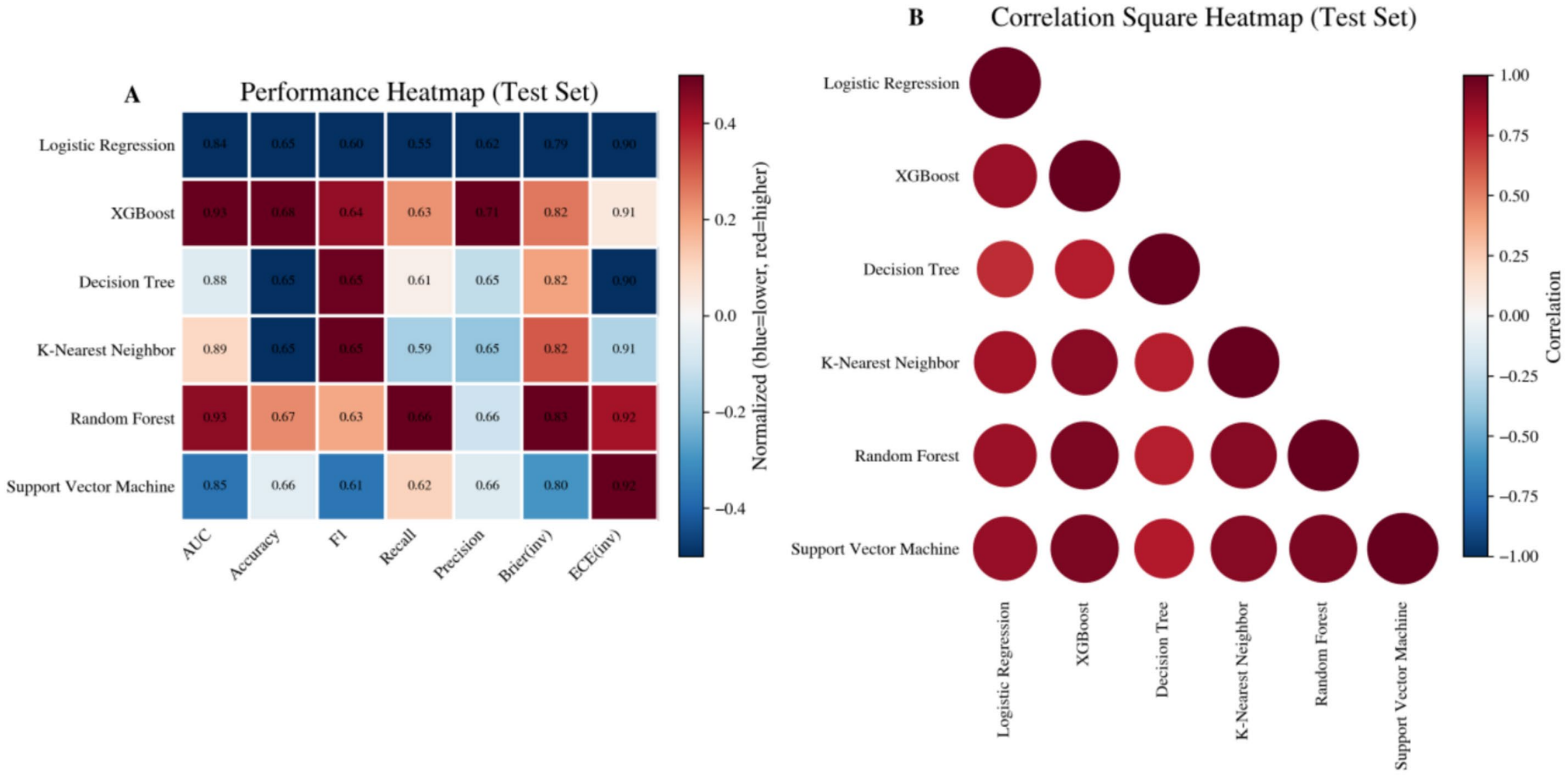
Heatmap summarizing prediction performance across models in the test set. **(A)** Performance heatmap showing normalized evaluation metrics (AUC, accuracy, F1-score, recall, precision, Brier score, and ECE) across different prediction models. **(B)** Correlation square heatmap illustrating pairwise correlations of model prediction outputs in the test set.

The multi-criteria heat map of the test set clearly summarizes the overall rankings of all models in terms of various AUC, accuracy, sensitivity, specificity, and F1 scores, allowing for direct identification of trade-offs such as high discrimination but mediocre calibration, or good calibration but insufficient discrimination (Figure 5). Combining all three: Discrimination, Calibration, and overall Classification Quality, XGBoost emerges as the primary model for further interpretation and application.

### 5.3 Classification transparency and error profile

To improve the interpretability and auditability of the model’s decision-making process, this study reports the final selected model’s classification results and the distribution of error types in the test set (Table 7). From the confusion matrix, we can see that the model correctly identifies the majority of the TP (97) as low resilience students, with stable performance in differentiating normal/high-resilience students, resulting in TN = 299, indicating a balanced discriminatory ability for all samples (Table 7). On a metric level, the model achieved a sensitivity of 0.815 and a specificity of 0.837, indicating it can “identify high-risk students” without misclassifying normal students as low resilience (Table 7).

**Table 7:** Confusion matrix of the final selected model for classifying low psychological resilience in the test set.

A. Confusion Matrix			
**Actual Class**	**Predicted: Low Resilience**	**Predicted: Normal/High Resilience**	**Total**
Low Resilience	97 (TP)	22 (FN)	119
Normal/High Resilience	58 (FP)	299 (TN)	357
**Total**	155	321	476
B. Classification Metrics			
**Metric**	**Value**	**95% CI**	**Interpretation**
True Positive Rate (Sensitivity/Recall)	0.815	0.738–0.878	Correctly identified 81.5% of students with low resilience
True Negative Rate (Specificity)	0.837	0.795–0.874	Correctly identified 83.7% of students with normal/high resilience
Positive Predictive Value (Precision)	0.626	0.546–0.701	62.6% of predicted low-resilience cases were truly low
Negative Predictive Value	0.931	0.900–0.956	93.1% of predicted normal/high cases were truly normal/high
False Positive Rate	0.163	0.126–0.205	16.3% of normal/high resilience students misclassified as low
False Negative Rate	0.185	0.122–0.262	18.5% of low resilience students misclassified as normal/high
Accuracy	0.832	0.795–0.865	Overall correct classification rate
F1-Score	0.708	0.645–0.768	Harmonic mean of precision and recall
Matthews Correlation Coefficient	0.614	0.538–0.684	Balanced measure accounting for class imbalance

The model shows a high NPV (0.931), making the classification of normal/high resilience more trustworthy. On the contrary, the positive predictive value (PPV = 0.626) indicates that some “low resilience” samples have been misclassified as “low resilience,” necessitating secondary screening or stratified intervention in practice to avoid wasting resources (Table 7). The model attained an overall accuracy of 0.832, an F1-score of 0.708, and an MCC of 0.614, performing well in risk screening while remaining robust to class imbalance (Table 7).

### 5.4 Explainability and theory alignment of predictors

**Figure 6:**
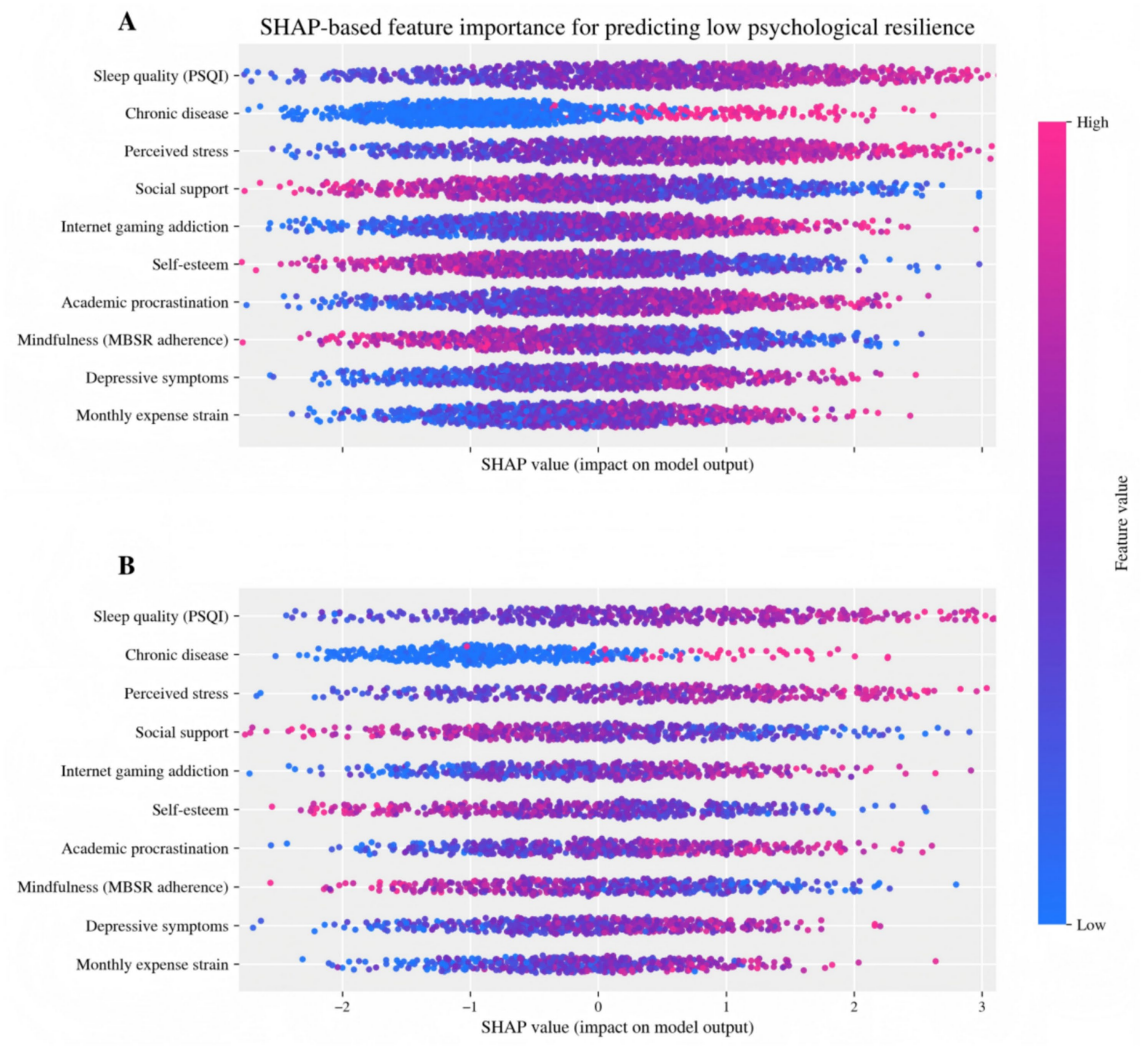
SHAP-based feature importance for predicting low psychological resilience: **(A)** training set; **(B)** test set.

To explain why the model made certain decisions, this study used SHAP to decompose the contributions of key predictors and their magnitude of impact (Figure 6), and stability checks were conducted separately on the training and test data. Overall, we observed extremely high agreement regarding the importances of the two datasets, suggesting that our model is not picking up on random patterns in the data and is instead identifying particular psychological and behavioral risk signals.

From the direction of feature contributions, sleep quality (PSQI), chronic disease, perceived stress, and depressive symptoms all contribute more to predictions of “low psychological resilience” when at higher levels, indicating the ongoing detrimental effects of physical and mental loads and negative emotions on resilience (Rodríguez-Planas et al., 2025). Conversely, resource-related variables such as social support and adherence to mindfulness training show a protective role in reducing the low-resilience risk at higher levels, reflecting the protective effect under the stress–resource perspective. Factors including online gaming addiction, academic procrastination, and self-esteem demonstrate both behavioral moderation and self-system resource explanations, indicating conceptual compatibility between the model’s outputs and the theoretical framework of the study. This facilitates the subsequent translation of predictive results into actionable, stratified intervention targets.

### 5.5 Clinical/practical utility for student support

**Figure 7:**
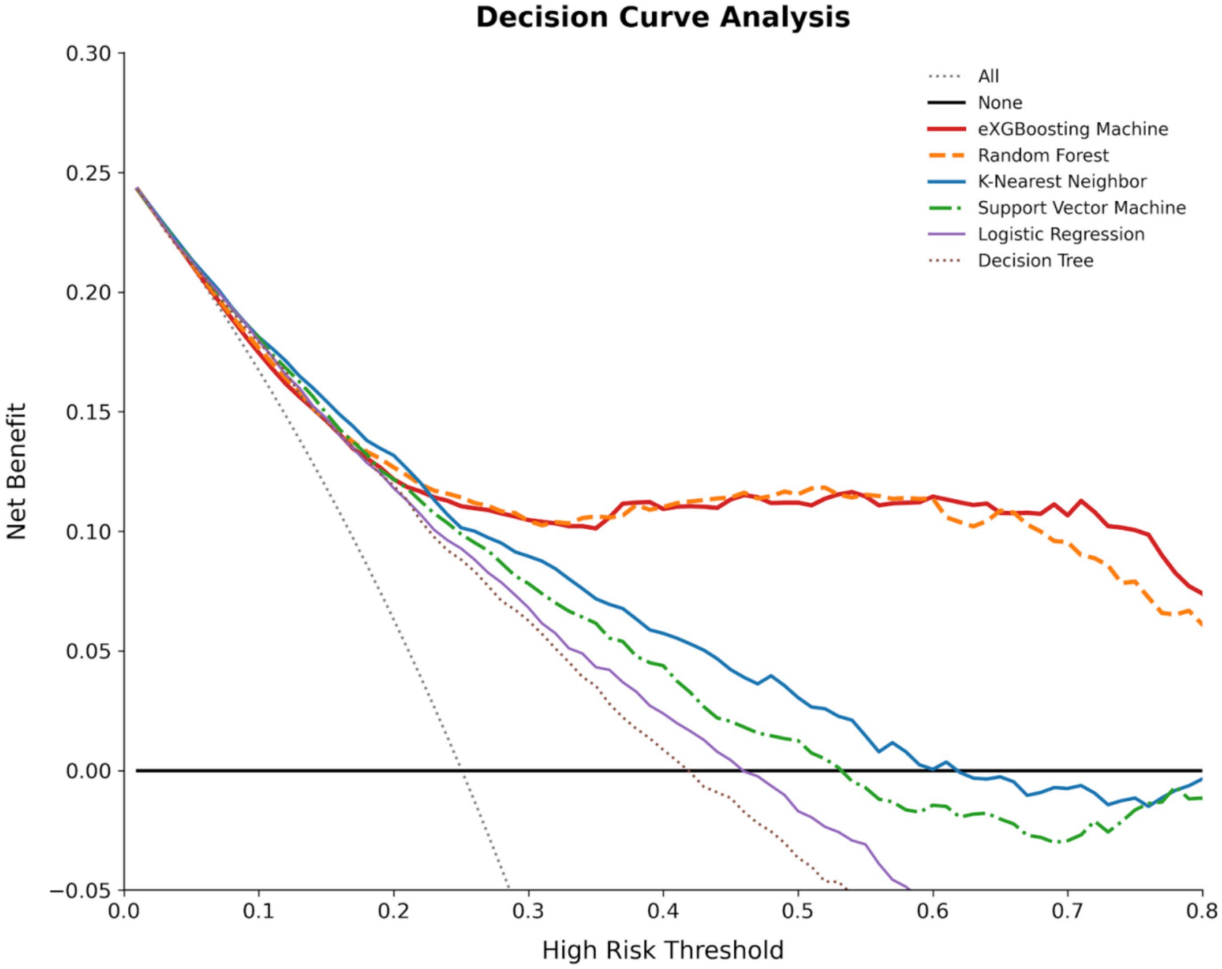
Decision curve analysis for each model in identifying students at risk of low psychological resilience.

To evaluate the model’s effectiveness in real-world student support decisions, this study employed Decision Curve Analysis (DCA) to compare net benefits across different risk thresholds (Figure 7). Overall, within a broad threshold range, the ensemble learning models (particularly XGBoost and Random Forest) consistently outperformed the ‘treat none’ and ‘treat all’ strategies, demonstrating higher net benefits than other models. This indicates their superior practical utility in the task of identifying students with low psychological resilience at high risk and initiating support services.

From an implementation perspective, DCA results indicate that schools can select models and trigger criteria with greater net benefits based on different risk thresholds when implementing tiered interventions (e.g., psychological screening reviews, counseling appointment prioritization, targeted sleep and stress management courses) under limited resources. This approach reduces ineffective interventions while improving coverage efficiency for genuinely high-risk students, thereby transforming predictive outputs into actionable student support strategies.

## 6 Integrated interpretation and reporting

This study employs a dual-track approach of “psychometric validation + machine learning prediction” to develop a low psychological resilience risk assessment framework for vocational college students. The results show that the resilience scale supports a three-factor solution of resilience, strength, and optimism, and demonstrates a well-fitting model. The scale achieves at least acceptable measurement equivalence across genders, indicating that measurement validity is consistent across gender groups. This serves as a stable basis for the following predictions.

In terms of reliability and validity, the scale is internally consistent regarding the sum and dimension scores. It is positively correlated with resources such as self-efficacy, social support, and emotion regulation, and negatively correlated with symptoms of depression, anxiety, stress, and academic burnout. These results support the theoretical expectations for psychological resilience as a resource for stress adaptation and demonstrate both construct and pragmatic validity (García-Pérez et al., 2025). In terms of predictive modeling, the test set results indicate that many models exhibit appropriate discriminative capabilities, with the Ensemble Learning model displaying the most robust results and consistent fit. Analyzing classification transparency shows that the final model achieves a balance between sensitivity and specificity; however, there are still some false positives and false negatives. This means that applications must be cautious about the thresholds they set and should manually verify results rather than relying completely on any single indicator’s interpretation.

The interpretable results indicate that factors such as sleep quality, chronic illness, feelings of stress, social support, online activities, self-esteem, academic procrastination, mindfulness, and sadness all tend to increase the risk of low resilience. This is in line with the “stress–resource” concept and provides actionable insights for tiered support at the school level. The decision curve also shows that the model exhibits net benefits within the commonly used threshold range, making it suitable for screening and prioritizing resources. However, it should not replace diagnostic conclusions, and limitations and generalizability boundaries must be stated in the report.

## 7 Discussion

Our findings provide strong empirical support for the theoretical framework outlined in the introduction—a multidimensional, theory-driven model of psychological resilience grounded in four interrelated domains: campus belonging, self-system resources, academic and occupational stressors, and health- and support-related behaviors. The successful development and validation of our XGBoost prediction model, coupled with SHAP interpretability analyses, confirm the relevance of these pre-specified dimensions and reveal how their components interact to shape resilience outcomes among vocational college students.

Crucially, the model’s high predictive performance suggests that psychological resilience is not merely the sum of isolated risk or protective factors, but rather an emergent property of dynamic interactions between stress exposure and available resources. For instance, perceived academic pressure (from the stressor domain) and social support (from the self-system domain) emerged as top predictors—but their influence is best understood not in isolation, but as part of a balancing act: resilience appears to hinge on the relative weight of stressors versus the accessibility and efficacy of coping resources. This finding offers robust, data-driven validation of the stress–resource theoretical perspective, demonstrating its explanatory power in a real-world educational context.

Moreover, our results carry significant theoretical implications beyond confirming expected predictors. Most notably, sleep quality—an indicator from the health behavior domain—ranked among the strongest predictors, even surpassing several psychosocial variables. This challenges purely cognitive or emotional conceptualizations of resilience and underscores the necessity of integrating physiological wellbeing into core resilience theory (Smith et al., 2025). It implies that resilience is fundamentally a whole-person phenomenon, wherein biological foundations (e.g., restorative sleep) enable or constrain the effectiveness of higher-order psychological processes. Consequently, theories of resilience must evolve to accommodate this biopsychosocial integration, moving beyond mental constructs alone.

Additionally, the superior performance of a machine learning model over traditional linear approaches hints at the presence of nonlinear effects and complex interactions among predictors—such as threshold effects or conditional dependencies (e.g., social support may buffer stress only when sleep quality is above a certain level). This suggests that resilience operates as a dynamic system rather than a static trait. Future theoretical models should therefore prioritize process-oriented, context-sensitive frameworks that can capture such complexity, potentially through computational or network-based approaches.

In summary, this study not only validates a structured, theory-informed framework for understanding resilience in vocational students but also advances resilience theory itself by (1) empirically demonstrating the centrality of the stress–resource balance, (2) expanding the construct to include physiological health as a foundational pillar, and (3) highlighting the need for dynamic, systems-based theorizing that accounts for nonlinearities and interactions. These insights pave the way for more holistic and effective interventions and more nuanced theoretical models in future research.

## 8 Conclusion

This study has successfully established and validated an AI-assisted, multi-dimensional psychological resilience evaluation system for vocational college students using a dual track approach that incorporates psychometric validation alongside machine learning prediction (Suhaimi et al., 2024). In addition to confirming the strong validity, reliability, and cross-ethnic measurement invariance of the three-factor structure (resilience, strength, and optimism) and establishing a measurement standard, it also showed that the multi-source feature-based machine learning model (especially XGBoost) had strong discriminative power (AUC = 0.883) and clinical net gain for identifying low levels of psychological resilience risk. The SHAP analysis identified the most important predictors, such as sleep quality, chronic disease stress, and social support, thus verifying the interpretability of the “stress–resource” theoretical framework in algorithmic decision-making (Avcı and Çınar, 2024). In summary, this integration of assessment serves as an important bridge linking psychological theories and data science applications, providing solid evidence and operational tools for vocational colleges to conduct early large-scale psychological risk screening of student groups, achieve precise stratification of students’ psychological resources, and offer customized interventions.

## Data Availability

The raw data supporting the conclusions of this article will be made available by the authors, without undue reservation.
